# Epidemiological study of penile cancer in a northeastern state - Brazil

**DOI:** 10.1590/0100-6991e-20233586-en

**Published:** 2023-10-30

**Authors:** Thais Cristina Loyola da Silva, Érika Gabrielle Pinheiro Ximenes, Ythalo Hugo da Silva Santos, Rodrigo Jerônimo Araújo, Eurides Araújo Bezerra de Macedo, Kleyton Santos de Medeiros, Irami Araújo-Filho

**Affiliations:** 1 - Liga Norte Riograndense Contra o Câncer Pesquisa e Inovação - Natal - RN - Brasil; 2 - Liga Norte Riograndense Contra o Câncer, Oncologia Clínica - Natal - RN - Brasil; 3 - Universidade Federal do Rio Grande do Norte, Departamento de Enfermagem - Natal - RN - Brasil; 5 - Universidade Federal do Rio Grande do Norte, Departamento de Cirurgia - Natal - RN - Brasil

**Keywords:** Penile Neoplasms, Carcinoma, Squamous Cell, Epidemiology, Penile Diseases, Surgical Oncology, Neoplasias Penianas, Carcinoma de Células Escamosas, Epidemiologia, Doenças do Pênis, Oncologia Cirúrgica

## Abstract

**Objective:**

to trace the clinical and epidemiological profile of penile cancer in Rio Grande do Norte/Brazil and relate them to data published in the literature.

**Methods:**

a cross-sectional study was conducted with 94 patients diagnosed with penile cancer in 2011-2018, treated at the Liga Norte Riograndense Contra o Cancer*.*

**Results:**

all patients were diagnosed with squamous cell carcinoma, mainly aged over 50 years, from the states interior, brown, illiterate, or with incomplete primary education. At diagnosis, 68% of patients were classified as having tumors =T2, and 30% had lymph node involvement. Distant metastases were detected in 2.1% of patients at diagnosis. Most patients received the diagnosis in the initial phase of the disease, but 20.2% were diagnosed in stage IV. Partial penectomy was the most performed surgery, and 10% of patients relapsed, mainly in the lymph nodes (87.5%). The mean follow-up of the patients was 18 months, with an estimated overall survival at five years of 59.1%. However, 25% of patients were followed up for up to 3 months, losing follow-up.

**Conclusion:**

the State of Rio Grande do Norte has a high incidence of penile cancer with a high frequency of locally advanced tumors at diagnosis and in younger patients younger than 50. Furthermore, socioeconomic factors interfere with early diagnosis and hinder access to specialized services.

## RESUMO

**Objetivo:**

traçar o perfil clínico e epidemiológico do câncer de pênis no Rio Grande do Norte/Brazil e relacioná-los com dados publicados na literatura.

**Métodos:**

realizou-se estudo transversal de 94 pacientes diagnosticados com câncer de pênis no período de 2011-2018, tratados na Liga Norte Riograndense Contra o Câncer.

**Resultados:**

todos os pacientes foram diagnosticados com carcinoma espinocelular, principalmente com idade acima dos 50 anos, provenientes do interior do estado, pardos, analfabetos ou com ensino fundamental incompleto. Ao diagnóstico, 68% dos pacientes foram classificados com tumores =T2 e 30% possuiam envolvimento linfonodal. Metástases à distância foram detectadas em 2,1% dos pacientes ao diagnóstico. A maioria dos pacientes recebeu o diagnóstico na fase inicial da doença, mas 20,2% foram diagnosticados em estádio IV. Penectomia parcial foi a cirurgia mais realizada e 10% dos pacientes recidivaram, principalmente para linfonodos (87,5%). A média de seguimento dos pacientes foi de 18 meses, apresentando estimativa de sobrevida global em 5 anos de 59,1%. No entanto, 25% dos pacientes foram acompanhados por até 3 meses, perdendo o seguimento.

**Conclusão:**

o Estado do Rio Grande do Norte apresenta elevada incidência de câncer de pênis com alta frequência de tumores localmente avançados ao diagnóstico, assim como em pacientes mais jovens, menores que 50 anos de idade. Outrossim, o fator socioeconômico interfere no diagnóstico precoce e dificulta o acesso a serviços especializados. .

## INTRODUCTION

Penile cancer is a rare neoplasm in the United States and Europe, accounting for less than 1% of cancers in men. However, it is more frequent in less developed areas, corresponding to 10-20% of all male malignancies^1^ ^,^ ^2^, clearly indicating the association of the pathology with local economic conditions^3^ ^,^ ^4^.

Brazil is one of the countries with the highest incidence of penile cancer in the world^5^ ^,^ ^6^, which may correspond to 2.1% of all neoplasms in men, being five times more prevalent in the North and Northeast regions^7^, where most penile amputations are performed^6^. 

Penile cancer is an aggressive and mutilating disease that affects self-esteem, with psychological and functional repercussions, which make rehabilitation and social reintegration difficult^7^. Despite being a complex surgery, studies show that penile reconstruction (phalloplasty) is possible after penectomy when the functional length of the penis is inadequate for a man to urinate standing up or have sexual intercourse^8^ ^-^ ^10^.

Its risk factors are multiple, but its carcinogenesis is not entirely elucidated^7^ ^,^ ^11^. Age greater than 60 years, phimosis, poor personal hygiene habits, smoking, history of sexually transmitted diseases, mainly human papillomavirus (HPV), are the factors most described in the literature, in addition to low educational level and limited access to health services^3^ ^,^ ^4^ ^,^ ^7^ ^,^ ^12^.

Therefore, the Brazilian Penile Cancer Consensus argues that reducing the incidence of this cancer is possible by encouraging intimate hygiene education, neonatal circumcision, smoking cessation, vaccination against HPV among young people, and the use of condoms^4^.

Late diagnoses are based on the male chauvinist culture that self-care with men’s health configures a role of passivity, dependence, and male fragility, primarily when related to the genital organ. This thought is predominant in northeastern Brazil^3^ ^,^ ^13^.

Unlike women, social stigma generally makes men seek fewer health services or seek them out in situations of already manifest disease, accidents, or injuries, which generates underreporting of cases. Furthermore, there is the fear of being diagnosed with cancer, commonly associated with death, painful treatments, and mutilations^3^ ^,^ ^13^.

Nursing has crucial participation in the direct care of individuals and the health education of a community, mainly through the Family Health Strategy established by the Unified Health System (SUS), through which it can actively act in the promotion, prevention, and the self-care of Men’s Health, or even bring it closer to the strategy with family support to instruct how to prevent penile cancer, diagnose it early and have better prognoses^14^.

Penile neoplasia usually presents as a verrucous, flat or ulcerated skin lesion in the genital region. Its diagnosis is made through wide and deep biopsy of the lesion to evaluate the histology and the degree of cell differentiation^4^ ^,^ ^15^ ^,^ ^16^.

The predominant histological types are squamous cell carcinoma (SCC), melanoma, lymphoma, sarcoma and basal cell carcinoma. The prognosis of the disease depends on the stage of the neoplasm (favorable <T1), the volume of the lesion (favorable <2cm), the degree of cell differentiation and the presence of vascular/lymphatic invasion in the primary lesion (metastases are more frequent when there is microvascular invasion)^4^ ^,^ ^15^ ^,^ ^16^.

Prospective epidemiological studies on penile cancer are scarce and small. And, despite the high incidence, Brazil has few studies on the subject. Then, the guiding problem of the study arose: What is the epidemiological and clinical profile of penile carcinoma in the Potiguar territory?

In this regard, when considering the taboo related to diseases involving the male genital organ, it is essential that each state traces the epidemiological profile of penile cancer so that the topic is more discussed in society, men realize the importance of early diagnosis and, thus, data close to the real are obtained regarding the prevalence of the disease in the state and country and the male population is benefited.

The main objective of the present study was to outline the clinical and epidemiological profile of penile cancer in the Potiguar territory and relate them to data published in the literature.

## METHODS

A retrospective cross-sectional study was carried out following the verifications of the STROBE Guidelines^17^. The study sample consisted of 94 patients diagnosed with penile cancer from January 2011 to December 2018 and treated at the Liga Norte Riograndense Contra o Cancer. It is a highly complex oncological center in Rio Grande do Norte and the main reference center for the treatment of penile cancer in the state, whose population is 3,168,027 inhabitants, according to data from the last census, carried out in 2010 by the Brazilian Institute of Geography and Statistics (IBGE). Data were obtained from the collection of data from physical and electronic medical records of all patients with an anatomopathological diagnosis of penile cancer. The variables studied were age, color, education level, origin, histological type, tumor grade, TMN AJCC 8^th^ Edition system staging, type of surgery, lymphadenectomy, chemotherapy, radiotherapy, and palliative treatment. Data were stored in the Microsoft Excel spreadsheet editor. The software SPSS 24 for Windows (Statistical Package for Social Sciences; IBM, USA) was the computational resource used, in which exploratory data analysis, association tests between variables, and overall survival analysis were performed. Fisher’s Exact Test was used to verify associations between variables. Survival analyses were performed using the Kaplan-Meier method and the Log-Rank test to verify the existence of significant differences in the estimated curves. The significance threshold was p≤0.05 with a 95% confidence interval. The age-standardized incidence was calculated using the standard world population proposed by Segi and modified by Doll et al.^18^. The Cancer Incidence in the Five Continents method of the International Agency for Research on Cancer (IARC) was also applied, in which the number of cases in each five-year age group was divided by the size of the population in each age group. As other smaller cancer centers receive patients with penile cancer in Rio Grande do Norte, the estimated incidence from the data presented here provides an approximate estimate of reality. The study was carried out by the principles of the Declaration of Helsinki and approved by the Research Ethics Committee of the Liga Norte Riograndense Contra o Cancer [process number 02089218.6.0000.5293], and informed consent was waived.

## RESULTS

From 2011 to 2018, 94 patients were diagnosed with penile cancer, resulting in an average of 11.7 new cases yearly. The age-standardized incidence recorded in this study was 6.38 cases/100,000 inhabitants (Table 1), exceeding previously published rates. 

**Table 1:** Age-standardized incidence of patients diagnosed in Liga Norte Riograndense Contra o Cancer.

Age Group	Stardard Population (a)	Incidence (b)	Male Population in the RN (c)	Gross Incidence Rate	e = a*d
0 - 4	12,000	0	120,553	0,00000	0,0000
5 - 9	10,000	0	130,579	0,00000	0,0000
10 - 14	9,000	0	149,689	0,00000	0,0000
15 - 19	9,000	0	149,871	0,00000	0,0000
20 - 24	8,000	1	155,051	0,00001	0,0516
25 - 29	8,000	0	142,913	0,00000	0,0000
30 - 34	6,000	4	124,136	0,00003	0,1933
35 - 39	6,000	2	108,483	0,00002	0,1106
40 - 44	6,000	5	103,637	0,00005	0,2895
45 - 49	6,000	6	91,203	0,00007	0,3947
50 - 54	5,000	6	68,712	0,00009	0,4366
55 - 59	4,000	13	52,702	0,00025	0,9867
60 - 64	4,000	12	47,273	0,00025	1,0154
65 - 69	3,000	13	34,185	0,00038	1,1409
70 - 74	2,000	11	27,411	0,00040	0,8026
75 - 79	1,000	7	17,196	0,00041	0,4071
80+	1,000	14	25,293	0,00055	0,5535
Total	100,000	94	1,548,887		6,3824

*Standard population suggested by Segi (1960) and modified by Doll et al. (1966). **RN population based on the 2010 Demographic Census. Source: Authors (2022).

According to the analysis, 72.2% of the patients came from the state’s interior, while 27.8% came from the capital (Table 2). The majority, 67.8%, were brown (Table 2).

**Table 2:** Distribution of cases, according to origin, age group, skin color, education, TNM classification, clinical staging, surgical treatment, chemotherapy and radiotherapy treatment and relapse (%), of patients diagnosed in the Liga Norte Riograndense Contra o Câncer.

Variable	%	Variable	%
Origin		Age Group	
Natal	27.80	≤50 anos	18.56
Interior	72.20	≥50 anos	81.44
Skin Color		Education	
Brown	66.70	Illiterate	30.90
White	23.70	Elementary Incomplete	53.20
Black	9.70	Elementary Complete	6.40
		High School Incomplete	1.10
		High School Completo	6.40
		Graduated	2.10
TNM			
Tis	1.10	Nx	22.30
T1a	28.70	N0	47.90
T1b	2.10	N1	7.40
T2	37.20	N2	4.30
T3	28.70	N3	18.10
T4	2.10	M0	97.9
		M1	02.1
Clinical Staging		Surgical Treatment	
Tis	1.10	Partial Penectomy	48.90
I	21.30	Partial Penectomy with Radical Inguinal Lymphadenectomy	20.20
IIA	27.70	Total Penectomy	9.60
IIB	19.10	Unilateral Lymphadenectomy	23.60
IIIA	7.40	Bilateral Lymphadenectomy	76.40
IIIB	3.20		
IV	20.20		
Treatment with Chemotherapy or Radiotherapy		Recurrence (10%)	
Exclusive Adjuvant Chemotherapy	5.40	Lymph Nodes	87.50
Exclusive Adjuvant Radioterapia	3.30	Lung and Bones	12.50
Adjuvant Chemoradiotherapy	2.10		

Source: Authors (2022).

Similar to data from the world literature, most patients (84.1%) were illiterate or had incomplete primary education, and 81.4% were diagnosed with penile cancer aged 50 years or older (Table 2). The mean age at diagnosis was 63 years.

All patients were diagnosed with squamous cell carcinoma, 80.9% of which had histological grade 2. Angiolymphatic invasion and perineural invasion, factors known to have a worse prognosis^19^, were present in 87.7% and 83% of cases, respectively.

Risk factors related to penile cancer, such as phimosis, smoking, alcoholism, history of sexually transmitted diseases, human papillomavirus (HPV) infection, and presence of pre-neoplastic diseases, could not be evaluated the study due to a deficit in filling out medical records.

By the AJCC 8^th^ edition TNM classification system, 68% of the patients were classified as ≥T2 (Table 2). Lymph node involvement at diagnosis was present in 30% of patients and distributed as follows: N1 in 7.4%, N2 in 4.3%, and N3 in 18.1% (Table 2), and distant metastases were detected in 2.1% of patients at diagnosis, with bones and lungs being the main sites affected. 

Most patients were diagnosed in the early stages of the disease, in clinical stages I (21.3%), IIA (27.7%), and IIB (19.1%). 20.2% of the disease was diagnosed in stage IV (Table 2).

Regarding surgical procedures, approximately half of the patients (48.9%) underwent partial penectomy exclusively, 20.2% partial penectomy with radical inguinal lymphadenectomy, and 9.6% total penectomy. Among the patients who underwent lymphadenectomy, 76.4% underwent bilateral lymphadenectomy, and 23.6% underwent unilateral lymphadenectomy (Table 2).

About 5.4% received adjuvant chemotherapy alone, and 3.3% received adjuvant radiotherapy alone. At the same time, 2.1% underwent adjuvant chemo and radiotherapy. Furthermore, 10% of patients relapsed, mainly in lymph nodes (87.5%), lungs, and bones (12.5%).

**Figure 1:**
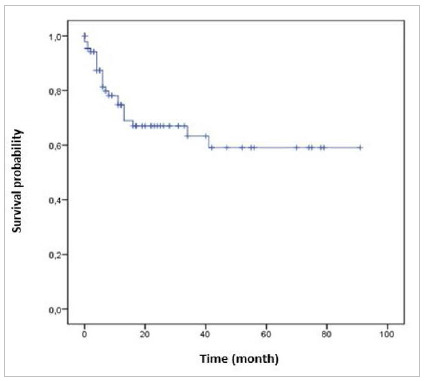
Overall survival estimated by Kaplan-Meier, in 5 years, of patients diagnosed in the Liga Norte Riograndense Contra o Cancer.

Mortality significantly worsened with staging. While 12.5% of patients in stage I died, 33.3% of patients in stage IV died in the same period (Table 3). The mean follow-up of the patients was 18 months, with an estimated overall survival at five years of 59.1% (Figure 1). However, 25% of patients were followed for up to 3 months.

**Table 3:** Association between staging at diagnosis and mortality of patients followed by Liga Norte Riograndense Contra o Cancer.

Clinical Staging	Death				p-value
	Yes		No		
I	3	12.5%	17	24.6%	0,01
II	7	29.2%	37	53.6%	
III	6	25.0%	4	5.8%	
IV	8	33.3%	11	15.9%	
Total	24	100.0%	69	100.0%	

Source: Authors (2022).

The lack of data collection records makes it impossible to better elucidate the epidemiological profile of cancer.

## DISCUSSION

One of the world’s highest incidences of penile cancer is found in India, with rates of 3.32/100,000 habitants, and the lowest is in Jews born in Israel, with an index close to zero, which is related to neonatal circumcisions^7^ ^,^ ^20^.

In Brazil, the general relative incidence is 2.1% of male neoplasms, reaching 5.7% in the Northeast region, 5.3% in the North region, 3.8% in the Midwest region, 1.4% in the Southeast region, and 1 .2% in the South region^7^.

In this study, 72.2% of patients come from the state’s interior, and 84.1% are illiterate or with incomplete primary education, presenting epidemiological characteristics like those described in other studies, especially those from developing regions whose socioeconomic reality is identical to that of Rio Grande do Norte State^1^ ^,^ ^2^ ^,^ ^21^ ^-^ ^23^.

Unfortunately, the risk factors related to penile cancer could not be evaluated in the study due to a lack of data in medical records, which constitutes a constructive criticism of a complete patient anamnesis and history, not only for scientific collaboration but for better and comprehensive understanding and care for the patient, serving as a warning to health institutions about the importance of this^13^ ^,^ ^24^ ^-^ ^26^.

Even so, we agree with the literature regarding the participation of phimosis, lack of adequate intimate hygiene, and smoking in develop and aggravation of penile cancer^3^ ^,^ ^4^ ^,^ ^13^ ^,^ ^20^.

Although penile cancer is more frequent in the sixth decade of life^11^ in this study, 18.5% of patients were diagnosed under 50. The occurrence of this neoplasm in earlier age groups serves as an alert to the importance of its research in younger patients with suspicious lesions^4^ ^,^ ^20^.

Of note, 68% of patients were classified as T2 or higher, and 30% with lymph node involvement at diagnosis. These data are worrying, as it is well established that the advanced stage strongly correlates with the degree of invasion and the likelihood of regional and systemic metastases, leading to a worse prognosis for these patients^7^ ^,^ ^26^. 

Results of the analysis of the primary tumor were like those of Brazilian studies presented by Coelho et al. (2018) (66.4%), Favorito et al. (2008) (57.9%), and Couto et al. (2014) (63.6%). However, these data are higher than those found in studies in developed countries, such as the United States (45.9 and 50.6%)^19^ ^,^ ^22^.

The scarcity of publications reflects the low incidence of the disease in rich countries, and consequently, most of the works come from isolated institutions and with small casuistry. Thus, relevant questions regarding the clinical management of penile cancer remain open, including, until now, we do not have a Brazilian consensus^5^.

As for the treatment, more than 90% of the patients underwent some surgical treatment with total or partial penectomy with or without inguinal lymphadenectomy. Furthermore, approximately 25% of patients were diagnosed with locally advanced or metastatic disease. These data demonstrate late diagnosis and delay in referral to specialized services, leading to more mutilating surgeries and palliative treatments^5^ ^,^ ^10^ ^,^ ^13^.

None of the patients underwent neoadjuvant chemotherapy, a treatment reserved for adjuvant or palliative purposes only, although 18% of patients had classification N3 at diagnosis. The main chemotherapeutic agents used were cisplatin and 5-fluorouracil. Platinum-based triple regimens, until then considered standard, were used in isolated cases^23^ ^,^ ^24^.

Lymph node involvement was present in 30% of the patients. However, only 6.5% underwent adjuvant radiotherapy. In unresectable local or locoregional recurrence cases, the most used treatment was radiotherapy with or without radiosensitizing chemotherapy. About 10% of patients relapsed, and 87.5% of cases for lymph nodes. Studies show that lymph node recurrence is one of the main factors of a worse prognosis in penile cancer^25^ ^,^ ^26^.

In Table 2, we observe that mortality significantly worsened with staging. In that study, which had an average follow-up of 18 months, 12.5% of patients in stage I died, while 33.3% of patients in stage IV died in the same period, which more than doubled. 

In addition, the socioeconomic and cultural profile of the carriers corroborates the fact that 25% of them were lost to follow-up after three months of diagnosis.

This neoplasm mainly affects men of low social class and education level, whose access to the reference health service is more complex. When they get medical care again, local and loco-regional recurrences are common^14^.

Although the Liga Norte Riograndense is a philanthropic institution and has more than 90% of its patients from the public service network and, theoretically, the data from this study are overestimated for this population, this disease tends to affect patients with more precarious socioeconomic conditions, which makes early diagnosis and access to specialized services difficult^4^ ^,^ ^14^.

To change this devastating scenario, national campaigns are essential, especially in the North and Northeast, to educate the population about this currently unknown neoplasm and alert Brazilian authorities about the importance of the topic. 

In addition, the training of health professionals themselves and a diagnosis and treatment flowchart should also be prioritized, since in addition to the lack of knowledge related to the disease, the patient also finds it difficult to be diagnosed and obtain adequate follow-up and treatment, mainly through the SUS network (Sistema Único de Saúde)^13^ ^,^ ^14^.

Nursing, within its competencies, plays a fundamental role in the promotion, prevention, and self-care of Men’s Health, emphasizing primary care, where qualified listening is more present, and it is possible to interact, raise awareness and educate the community^14^.

## CONCLUSION

Considering only data from a single treatment center, Rio Grande do Norte has a high incidence of penile cancer with a high frequency of locally advanced tumors at diagnosis and in patients younger than 50 years. Since American and European studies describe penile cancer as a rare neoplasm.

Due to the importance of Brazil in the world scenario of penile cancer, it is necessary to implement measures that allow prevention, early diagnosis and less agressive treatment, in addition to the role of leading clinical research to better understand carcinogenesis and obtain more effective therapies.
